# Interaction Regulation Between Ionomer Binder and Catalyst: Active Triple‐Phase Boundary and High Performance Catalyst Layer for Anion Exchange Membrane Fuel Cells

**DOI:** 10.1002/advs.202101744

**Published:** 2021-08-02

**Authors:** Huixing Cao, Ji Pan, Hairong Zhu, Zhe Sun, Bowen Wang, Junliang Zhao, Feng Yan

**Affiliations:** ^1^ College of Chemistry Chemical Engineering and Materials Science Soochow University Suzhou 215123 China

**Keywords:** alkaline stability, catalytic activity, fuel cells, ionomer binder, structural evolution

## Abstract

As one of the most crucial components, the catalyst layer (CL) plays a critical role in the performance of anion exchange membrane fuel cells (AEMFCs). However, the effect of the structural evolution of ionomer binder on the micromorphology and catalytic activity of CL is yet to be clarified. In this study, pyrrolidinum and quaternary ammonium cations are attached to the polyphenylene oxide (PPO) backbone through flexible spacer units (five, seven, or nine carbon atoms) with different terminal alkyl groups. The Van der Waals force and electrostatic repulsion between the ionomer binder and catalyst are regulated through the flexible spacer units and terminal alkyl groups to alleviate the agglomeration of catalyst particles and acquire a high catalytic activity. To evaluate the electrochemical stability of the cationic groups, the alkaline stability of the ionomer binder is tested under a constant voltage to simulate the true operational environment of the fuel cells. The results reveal that the degradation of the cation groups of ionomer binder is accelerated under a constant voltage condition. This phenomenon in neglect earlier, may serve as a useful reference for the synthesis and performance enhancement of ionomer binders.

## Graphical abstract

Reasonable interaction regulation between the catalyst and ionomer binder forms a high performance catalyst layer. Here, the multiple interactions, including the Van der Waals force and electrostatic repulsion between the ionomer binder and catalyst, are regulated through the flexible spacer units and terminal alkyl groups, which alleviate the agglomeration of catalyst particles and acquire a high catalytic activity.

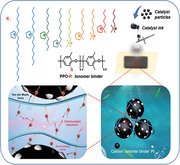

## 1 Introduction

With the rapid aggravation of global energy crisis and environmental pollution caused by fossil fuel combustion, the development of clean, efficient, and environment‐friendly energy conversion devices has garnered considerable attention. Fuel cells (FCs) are one of the most efficient and environment‐friendly energy conversion devices with almost zero harmful emissions, which can be used to process a variety of fuels.^[^ ^1^, ^2^, ^3^, ^4^ ^]^ Compared with the mature proton exchange membrane fuel cells (PEMFCs), alkaline anion exchange membrane fuel cells (AEMFCs) exhibit various advantages; one of the most remarkable advantages is their excellent electro‐kinetics for oxygen reduction reaction (ORR).^[^ ^5^, ^6^ ^]^ Consequently, AEMFCs exhibit immense potential to use a non‐precious metal as a general catalyst in the future to realize large‐scale energy conversion for practical applications.^[^ ^7^, ^8^, ^9^ ^]^ Nevertheless, developing a non‐platinum group metal electrocatalyst for hydrogen oxidation reaction (HOR) in alkaline medium is challenging, and a good candidate has not been identified yet.^[^ ^10^ ^]^

As an essential component of AEMFCs, the catalyst layer (CL) formed by an ionomer binder and catalysts is sandwiched between the ion exchange membrane and gas diffusion layer to form a membrane electrode assembly (MEA). During the catalytic reaction process, the fuel (gas), ion‐transportation medium water (liquid), and catalyst particles (solid) interweaved in the CL form a complex triple‐phase boundary (TPB), which dominates the rate of ion, electron, and mass transportation.^[^ ^11^, ^12^ ^]^ In fact, it is difficult to obtain a homogeneous catalytic system in CL. Generally, commercial platinum catalyst particles are attached to the carbon base, which easily agglomerate to spontaneously form ionomer microstructure during the solidification.^[^ ^13^ ^]^ In this case, the catalyst particles are partially encapsulated by the aggregates of ionomer binder and surrounding water clusters, and an alienated ion‐conducting phase may be formed in the MEA, which restricts the catalytic activity and ion transportation rate.^[^ ^14^ ^]^ Therefore, the ionomer binder should act as a good dispersant and the interaction between the ionomer binder and the catalyst particles should be optimal, which can not only prevent the catalyst agglomeration but also ensure the rapid transportation of active substance in the TPB.

However, the research on the ionomer binders of AEMFCs is still in a nascent stage. Xu et al. proposed an ionomer cross‐linking immobilization strategy to inhibit the agglomeration of catalyst particles and fabricate durable CL in MEAs for long‐term application in AEMFCs.^[^ ^15^ ^]^ He and coworkers proposed a structural engineering strategy to immobilize a catalyst on the side chain of the ionomer binder to obtain a composite material, which facilitated a homogeneous catalysis environment inside the ion‐flow channels that greatly improved the mass transfer and turnover frequency.^[^ ^16^ ^]^ Peng et al. used operando neutron imaging and X‐ray computed tomography in combination with electrochemical test to understand the distribution of water in AEMFCs for intelligent water management in the CL.^[^ ^17^ ^]^ Zheng and coworkers found that the CO_3_^2–^ anions were transported through the AEM to the anode by migration, leading to the carbonation of the ionomer, which in turn reduced the cell performance.^[^ ^18^ ^]^ In most cases, the ionomer binder used in AEMFCs is not synthesized independently, and the same off‐the‐shelf structure is selected as the membrane.^[^ ^19^, ^20^ ^]^

Until now, the interaction between catalysts and ionomer binder is still not clear due to the lack of systematic analysis. Based on previous research, the interaction between adsorbates and catalyst substrates is quite complex. Yuan et al. used density functional theory (DFT) to show that due to the Van der Waals (VdW) force provided by the adsorbates, noticeable charge redistribution happens on the surface of the catalyst, which may restrict the catalytic activity of the catalyst.^[^ ^21^ ^]^ Matanovic and co‐workers suggested that when the aromatic polymer is used as the ionomer binder in AEMFCs, the interaction between metal catalyst and ionomer binder in the CL is mainly decided by the adsorption of benzene on the catalyst surface, which determines the adsorption energy and catalytic activity of the CL.^[^ ^22^ ^]^ Meanwhile, the electrostatic repulsion between the ionomer binder and metal catalyst possibly affects their adsorption interaction, which decides the micromorphology of the CL.^[^ ^23^ ^]^ The structure of the ionomer binder includes a polymer backbone and cationic groups, which impose VdW force and electrostatic force on the catalyst, thereby deciding the catalytic activity and micromorphology of the CL. However, the effect of the VdW force and electrostatic repulsion between the catalyst and ionomer binder on the CL has not been clarified yet. Herein, we show that the interaction between ionomer adsorbates and catalyst substrates can be regulated by the structural evolution of the ionomer binder to form a homogeneous catalytic system for exposing more active sites on the CL, which may play a vital role in the electrocatalytic activity of CL.

In this study, we prepared a series of ionomer binders with different locations of cationic groups in the side chain of polymer to realize a high‐performance CL. Then, the electrostatic repulsion is adjusted by tuning the charge density of cationic groups in the ionomer through the introduction of a series of alkyl chains with different lengths. The performance of all the ionomer‐binder‐decorated CLs is evaluated by the electrochemical ORR test, and the results indicate that the sample PPO‐7Py7@Pt/C exhibits the best catalytic activity and the maximum electrochemical surface area (ECSA). Besides, considering the operational environment of the ionomer binder, a novel method is established to assess the alkaline stability of cationic groups of the ionomer binder in the alkaline electrolyte solution under a constant voltage condition. Overall, the proposed structural evolution strategy and alkaline stability testing method for the ionomer binder play a critical role in improving the performance of CL, which can serve as a useful reference for the development of high‐performance AEMFCs.

## 2 Results and Discussion

### 2.1 Synthesis and Characterization of PPO‐Based Ionomer Binder

**Scheme 1:**
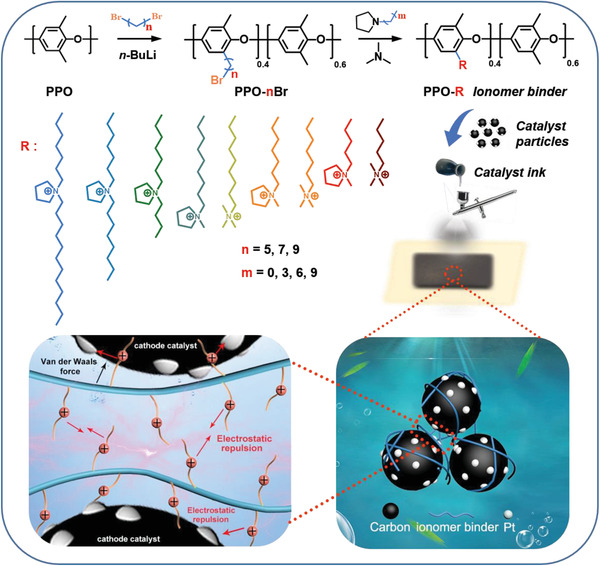
Schematic of the synthesis process of various ionomer binders using PPO backbone polymer, and the regulation mechanism of multiple effects between ionomer and catalyst. Here, the multiple interactions between catalyst and ionomer binder include the VdW force from ionomer backbone and the electrostatic repulsion from the cationic group, which are regulated to form a state‐of‐the‐art dispersion state in the catalyst layer of alkaline fuel cells.

To clarify the influence of the structural evolution of ionomer binder on the performance of the catalyst layer, pyrrolidinum (Py) and quaternary ammonium (QA) cations were attached to the polyphenylene oxide (PPO) backbone through flexible spacer units with different terminal alkyl groups, as shown in **Scheme** **1**. The chemical structure of PPO‐nBr and various ionomer binders was confirmed by ^1^H NMR spectra (Figures S6–9, Supporting Information). The IEC value of each ionomer binder was evaluated by Mohr titration (Table S1, Supporting Information).

### 2.2 Characterization of ORR Electrocatalytic Activity of Ionomer Binder‐Pt/C Catalyst

**Figure 1:**
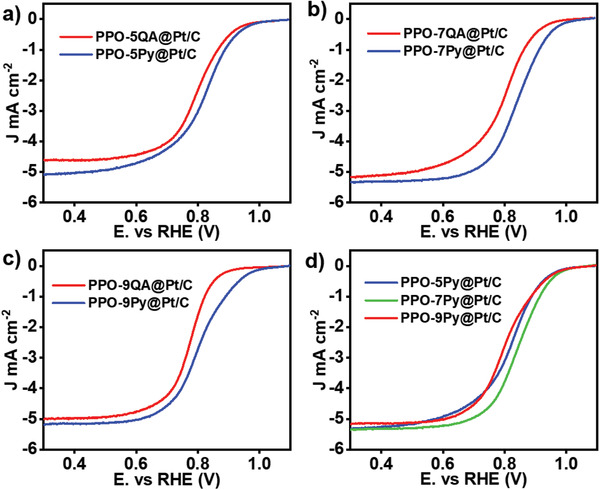
a–d) ORR polarization curves of PPO‐*n*Py/QA‐modified Pt/C catalyst (*n* = 5, 7, 9) recorded in O_2_‐saturated 0.1 m KOH aqueous electrolyte at a scan rate of 10 mV s^–1^. Pyrrolidine‐modified ionomer binder exhibits a superior electrocatalytic effect than QA‐linked ionomer due to the weaker electrostatic force. PPO‐7Py‐modified Pt/C sample exhibits a better electrochemical performance than the other samples, which can be attributed to the fact that a favorable VdW force imposed on the CL connects the ionomer binder and metal catalyst to form a good catalytic area.

During fuel cells operation, the rate of ORR is slower than that of HOR.^[^ ^24^ ^]^ Therefore, the sluggish kinetics of ORR are often flagged as a critical issue and focal area that needs to be resolved.^[^ ^25^, ^26^ ^]^A reasonable combination between the catalyst particles and ionomer binder to form a homogeneous catalysis area in the MEAs is a prerequisite to realize a high ORR rate at the cathode. To obtain an optimal ionomer binder, the ORR electrocatalytic activity of each ionomer binder‐Pt/C catalyst was examined by electrochemical test. The ionomer binder containing Py cationic group exhibits a superior electrochemical performance than that of QA cationic group, as proved by the positive variation of half‐wave potential (E_1/2_) and onset potential (0.1 mA cm^−2^) in **Figure** **1**a–c.^[^ ^27^ ^]^ This may be attributed to the lower charge density of Py ring cationic group compared with the QA cationic groups; the lower charge density provides a weaker electrostatic repulsion between the catalyst and ionomer binder, which is suitable for dispersion. Moreover, when a designated Py ring cationic group is installed in the polymer side chain, the ionomer possessing proper side chain (seven carbon atoms between the backbone and cation group) exhibits a preferable electrochemical behavior when it combines with catalyst particles. Further, it is obvious that the ORR activity improves initially and then decreases as the cation group moves far away from the backbone, as shown in Figure 1d.

The results indicate that the cation group species and the position of ionic segments in the ionomer govern the catalytic activity of the CL. The effect of cation group can be qualitatively understood by its charge density, which provides different electrostatic repulsion to the metal catalyst particles. The positive charge density of the nitrogen atom on the QA cations is higher than that on Py cations, causing a strong electrostatic repulsion between the metal catalyst and the positively charged cation groups. The strong static interaction separates the metal catalyst from ionomer binder and breaks their cohesive combination, thereby weakening the activity of CL. Regarding the cation group, if it is close to the polymer backbone, it is significantly affected by the VdW force that is mainly provided by the backbone. This intrinsic force induces strong adsorption of ionomer binder on the metal catalyst, which would block more surface site in electrode and cations may be oxidized on platinum electrode surface at high potential.^[^ ^28^ ^]^ Besides, when a benzene ring exists in the backbone, it can be easily oxidized at a high potential because the phenyl group tends to be parallel to the catalyst surface when the strong interaction imposed on the plane structure is constrained by VdW force. This neutralizes the ammonium hydroxide and triggers partial phenol formation, which cannot be removed from the catalyst interface easily, thereby limiting the ORR performance.^[^ ^2^, ^29^ ^]^

To a certain extent, the designated cation group can be moved far away from the polymer backbone by introducing a long alkyl side chain between them. This unique approach can alleviate the influence of VdW, but an extremely long alkyl chain may cause self‐assembly of cations to form a lipid‐like bilayer structure connecting polar head groups (cation group).^[^ ^30^, ^31^ ^]^ The restricted positively charged cation group can interact with the electrolyte or the innermost anion layer, hindering the electron migration around the platinum particles, as suggested by Korntshev.^[^ ^32^ ^]^ Overall, the electron transfer can be blocked by an excessive length of the side chain in the cation group, which further limits the ORR rate. Therefore, an appropriate‐length alkyl side chain was chosen to isolate the cation group and polymer backbone for regulating the interaction between ionomer binder and catalyst metal. This strategy to find an optimum balance between the cation group and backbone in the ionomer binder to expose maximum catalytically active sites in the CL is verified in this article.

### 2.3 Alkaline Stability of Cation Groups

Meanwhile, the ionomer binder of the CL is not only attacked by OH^−^ in an alkaline electrolyte solution but also exposed to a complex electrochemical environment with a relatively high potential during the cell operation. However, the alkaline stability of cation groups in the ionomer binder under an electrochemical condition has been neglected in the existing literature. Compared to the backbone of ionomer binder, cation groups play a significant role in assisting the transport of anion ions. We have previously proved that the cations attached in the benzyl ring are easily attacked by OH^−^.^[^ ^33^, ^34^ ^]^ Therefore, a long alkyl chain is chosen in the cation group in this article.

**Figure 2:**
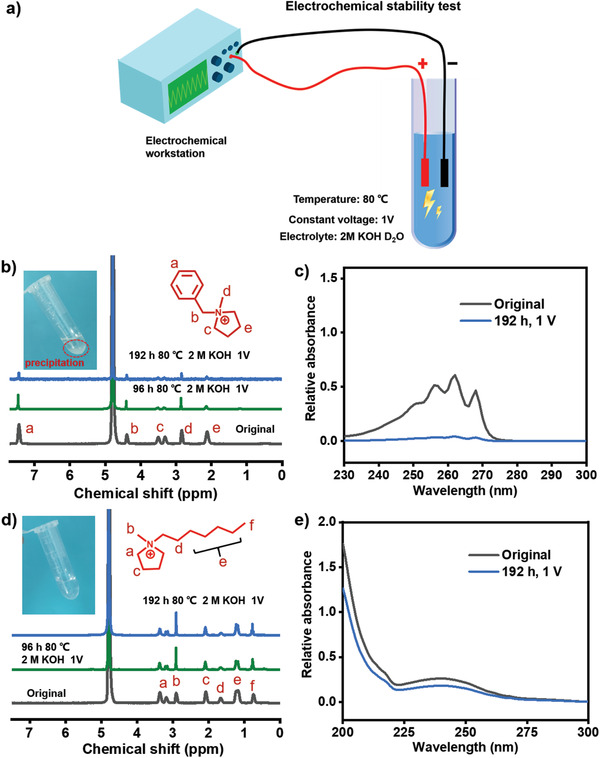
a) Schematic of electrochemical stability test. Compared with the traditional alkaline stability test, a constant voltage is applied to the basic electrolyte solution for examining the durability of cation group. b–e) NMR and UV spectra of benzyl–methyl pyrrolidine and *N*‐methyl‐1‐heptylpyrrolidine salt under a constant voltage condition in 2 m KOH D_2_O electrolyte at 80 °C. Precipitation appears and no obvious signal can be observed in the solution of benzyl–methyl pyrrolidine salt, which verifies that the sample is almost completely degraded. No evident variation can be observed in the *N*‐methyl‐1‐heptylpyrrolidine salt sample after the test, which proves that it maintains a stable state and is adequate for the cell operation.

To simulate the true operational environment of AEMFCs, for the first time, a constant voltage is applied to the base electrolyte solution to measure the alkaline stability of cation groups in CL, as shown in **Figure** **2**a. In contrast to the traditional alkaline stability test, the benzyl‐pyrrolidine and *N*‐methyl‐1‐heptylpyrrolidine [C_7_Py] cations salts are tested in 2 m KOH solution at 80 °C with a constant voltage of 1 V. The benzyl–pyrrolidine is completely degraded, which is accompanied by the formation of insoluble organic compounds (marked with a red circle). Further, comparing the nuclear magnetic resonance (NMR) and ultraviolet (UV) spectra, the peaks in both the spectra disappear after 192 h test, as shown in Figure 2b,c. However, the [C_7_Py] cations maintain a stable state, which is evidenced by no change in the NMR and UV spectra after the test, as shown in Figure 2d,e. These results demonstrate that the alkaline stability of the cations is affected due to the application of constant voltage, but the [C_7_Py] cations remain stable, which proves that the ionomer binders applied in this article are adequate for the cell operation. To examine the effect of the constant voltage, the stability of benzyl–pyrrolidine cations was separately tested in a 2 m KOH solution without applying any voltage and in a constant‐voltage non‐alkaline solution. The results show that the benzyl–pyrrolidine cations retain a steady‐state in the above test after 192 h, as evidenced by the NMR and UV spectra shown in Figure S11, Supporting Information, demonstrating that the constant voltage accelerates the alkaline degradation of the cation groups in the electrolyte. As Shaik et al. proposed, the external electric fields could lower the barrier height for the S_N_2 reaction.^[^ ^35^ ^]^ Therefore, the cation might be attacked more easily by OH^−^ with the effect of the electric field.

### 2.4 Hydrophobic Microphase Construction in the Catalyst Layer

**Figure 3:**
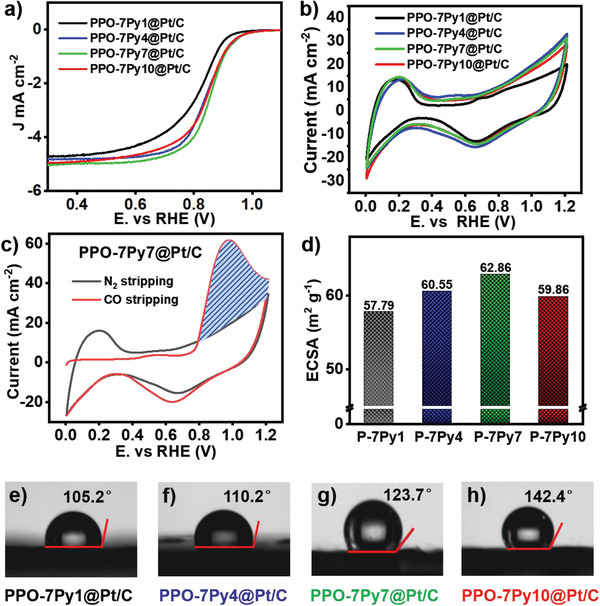
a) LSV curves of PPO‐7Py*m*‐modified Pt/C samples (*m* = 1, 4, 7, 10). PPO‐7Py7 modified sample exhibits the best electrocatalytic performance, as verified by the position of half‐wave potential. b) Cyclic voltammetry (CV) curves of PPO‐7Py*m*‐modified Pt/C sample. c) Evaluation of ECSA for the CL of PPO‐7Py7‐modified Pt/C sample based on CV measurement in N_2_‐saturated and CO‐saturated 0.1 m HClO_4_ solution. d) ECSA evaluation of the CL of PPO‐7Py*m*‐modified Pt/C sample. e–h) Water contact angle of the PPO‐7P*m*‐modified Pt/C sample, which confirms that its hydrophobic ability is enhanced due to the introduction of a long alkyl chain at the end of the cation group in the ionomer.

Besides, the efficiency of the CL can be enhanced by water management. The degree of ORR in aqueous electrolyte is hampered by water and oxygen. Most AEMFCs operate under a relative humidity (RH) of 100% to enable a sufficient water supply for the transfer of hydroxide ions, but the CL water management has been generally ignored. If there is excess water in the CL, a part of the inner filled nanopores used for oxygen permeation can be clogged and generate huge gas transportation resistance, which can decrease the ORR rate at the cathode.^[^ ^36^ ^]^ Although previously prepared long‐side‐chain ionomer binders modified by methyl–pyrrolidine cations have exhibited a decent electrochemical performance, their hydrophilic property might restrict the ability to remove water. With regard to water management in the MEA, a series of long alkyl chains were introduced, which not only acted as a hydrophobic section suspended on the cation group to enhance the capacity of water circulation on the CL surface but also adjusted the charge density of cations. **Figure** **3**a compares the ORR polarization of different ionomer‐modified samples tested in 0.1 m KOH aqueous solution. It is clear that the ionomers connected by a long alkyl chain exhibit positive shift with respect to that of the original sample in the linear sweep voltammetry (LSV) curve, which indicates a better electrochemical activity.

When a hydrophobic microenvironment is established at the interface between the aqueous electrolyte and metal catalyst, the nonreactive oxygenated species in the CL are drastically reduced due to the adequate oxygen flow in the internal space of the catalyst without retaining excessive water.^[^ ^37^, ^38^, ^39^ ^]^ Figure 3b shows the cyclic voltammetry (CV) curves of various ionomer‐modified samples, which reveal characteristic signals of proton hydrogen adsorption/desorption on the platinum catalyst. This indicates the capacity of hydrogen underpotential deposition (H_upd_), which can be observed in the potential range of 0.05–0.40 V. Further, it is found that all the ionomer–catalyst samples modified by long alkyl chain display a higher ECSA value than those without alkyl chain at the end of cation group, as proved by the measurement of the H_upd_ area.

Here, the ECSA values of various ionomer–catalyst samples were measured by CV curves in 0.1 m HClO_4_ aqueous solution with saturated N_2_ and CO separately, as proposed by Dekel et al.^[^ ^40^ ^]^ The CV curves of PPO‐7Py7 ionomer‐catalyst sample are taken as an example in Figure 3c to explain the assessment method of ECSA value. The boundary of the first cycle in Figure 3c is measured with an N_2_‐saturated aqueous electrolyte to avoid the influence of the external environment, and the second cycle area is correlated to CO stripping charge with active catalyst site adsorption (the shadow of blue solid lines label) after subtracting the standard baseline from the first cycle, which provides a more accurate result than the typical H_upd_‐adsorption approach.^[^ ^41^ ^]^ The CV curves of other ionomer–catalyst samples are shown in Figure S12, Supporting Information. And the estimated values of ECSA of various ionomer‐Pt/C samples are shown in Figure 3d. It is clear that the ionomer–atalyst sample modified by the hydrophobic long alkyl chain exhibits a larger ECSA value than the original ionomer, and the hydrophobicity of various samples is increased due to the carbon chain extension, which is confirmed by the measured water contact angle for each sample, as shown in Figure 3e–h. Interestingly, the heptyl‐decorated ionomer–catalyst sample exhibits the best electrochemical performance among all the samples. A plausible explanation for this phenomenon is that the suitable hydrophobic ability enhances the catalytic efficiency of cathode owing to the improvement in the ORR kinetics by the balanced water supply and removal of excess water.^[^ ^42^, ^43^ ^]^ If the surface of metal catalyst cannot hold sufficient water, the cathode may not maintain a regular ORR rate. Hence, the adjustment of water microenvironment around ionomer binder to optimize the catalyst operation should be focused on the CL. On the other hand, the charge density of cation group suspended in the side chain decreases due to the extension of alkyl chain. Therefore, the electrostatic repulsion between the metal catalyst and cation group is adjusted to obtain a proper adsorption interaction between ionomer binder and catalyst and realize a rational system in the CL.

### 2.5 Adsorption of Cation Groups on the Pt Surface

**Figure 4:**
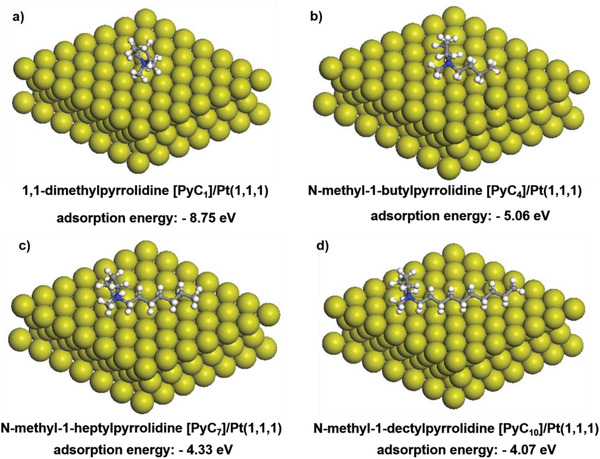
Adsorption energies (in eV) of a) 1,1‐dimethylpyrrolidine [PyC_1_], b) *N*‐methyl‐1‐butylpyrrolidine [PyC_4_], c) *N*‐methyl‐1‐heptylpyrrolidine [PyC_7_], and d) *N*‐methyl‐1‐dectylpyrrolidine [PyC_10_] cations on the Pt (111) surface, which are calculated using DFT method. The interaction between the catalyst and the ionomer binder tends to become more reasonable with the increase in the decorated alkyl chain and decrease in the adsorption energy.

The structure of the ionomer binder in CL, which includes the cation groups, can affect the interaction between the catalyst particles and ionomer binder. The impact of various cation groups with the adsorption energies on the surface of the Pt particles was investigated by DFT calculation to explain the varied interaction between the ionomer binder and catalyst based on a previous study.^[^ ^44^ ^]^ The adsorption energy of various pyrrolidine cation groups is calculated as −8.75, −5.06, −4.33, and −4.07 eV with the increase in the alkyl chain when it interacts with the Pt (1,1,1), as shown in **Figure** **4**. This implies that the adsorption energy between the catalyst and ionomer binder tends to decrease with the increase in the decorated alkyl chain. The electrochemical activity of the samples modified by the above cation groups is consistent with Sabatier's principle: an “ideal catalyst” should interact with the substrate neither too strongly nor too weakly.^[^ ^45^, ^46^ ^]^ Consequently, the faster transportation of oxygen, water, and active substrates can be realized in the TPB of CL due to the poor restrictive effect between the cation groups and the catalyst, which arises from weak and appropriate adsorption energy.

**Figure 5:**
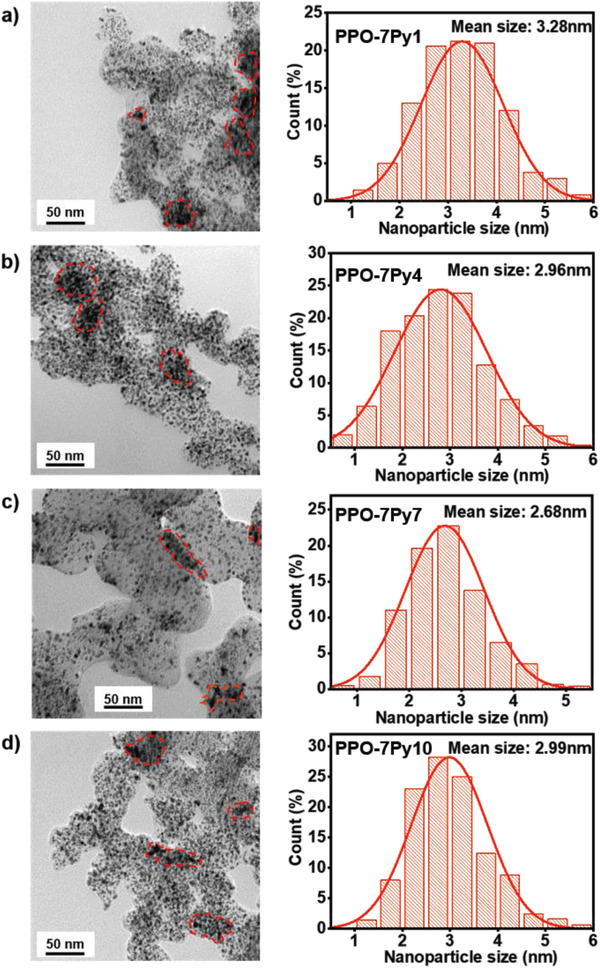
a–d) TEM images and particle size distribution of PPO‐7Py*m* (*m* = 1, 4, 7, 10) ionomer‐modified CL. The dispersion of each sample can be measured by the distribution and mean size of catalyst particles.

Based on the effective regulation of multiple effects and hydrophobic long alkyl chain decoration, the PPO‐7Py7‐modified catalyst sample presents the best dispersion state than the other specimens, as shown in the transmission electron microscopy (TEM) images in **Figure** **5**a–d. This is because the average size of Pt nanoparticles in PPO‐7Py7 ionomer‐modified CL is lower than that in the other samples, which indicates that the degree of agglomeration in this sample is inferior to that in the other samples. This in turn exposes more catalytically active sites at the TPB (the agglomerated area is marked by red lines), contributing to an adequate ECSA in the CL of cathode and providing a sufficient ORR area for AEMFC operation.

### 2.6 Thermal and Alkaline Stability of Ionomer Binders

**Figure 6:**
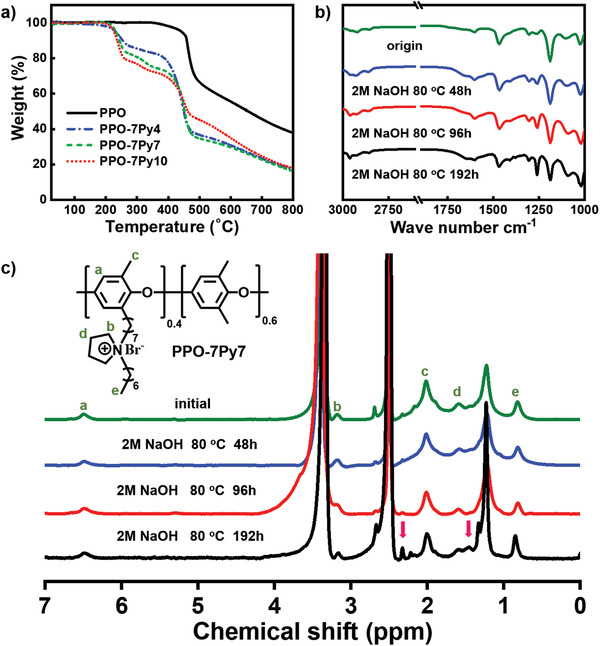
a) Thermogravimetric analysis (TGA) traces of native PPO and a series of long alkyl‐chain‐modified ionomers (PPO‐7Py4, PPO‐7Py7, and PPO‐7Py10). All the tested samples were measured under N_2_ at 10 °C min^–1^. The thermal degradation temperatures for each ionomer binder are above the operating temperatures of commonly used electrochemical devices. b) FTIR spectra of PPO‐7Py‐7 stored in 2 m aqueous NaOH solution at 80 °C after 0, 48, 96, and 192 h. Almost no change is observed in the spectra after the test, which proves that the sample maintains a steady‐state in an alkaline solution. c) ^1^H NMR spectra of PPO‐7Py‐7 stored in 2 m aqueous NaOH solution at 80 °C after 0, 48, 96, and 192 h. Only a minor change in the spectra is observed, and the signals of unknown origin are indicated with pink arrows. This indicates that the ionomer remains stable under practical operation.

The thermal decomposition temperature (*T*_d,95_, temperature at 5% weight loss) of the prepared hydrophobic alkyl‐chain‐modified ionomers was investigated. All the ionomer binders in the form of Br^–^ were found to decompose in two steps with different temperature areas (**Figure** **6**a). The native PPO without spacer and extender was decomposed at *T*_d,95_ = 441 °C due to the breaking of C—O bond in the backbone, while the ionomer binders with an extended side chain began to decompose at *T*_d,95_ = 231 °C (210 °C lower), where the non‐hydroxide pyrrolidine groups located in the modified PPO side‐chain started to break. Besides, the second decomposition temperature for the ionomer was nearly 427 °C close to the degradation temperature of native PPO. In any case, the thermal degradation temperatures for the ionomer were above the operating temperatures of typical electrochemical devices.

At the original area of the reaction, a huge amount of hydroxide ions is generated in the CL due to the ORR process during the cell operation. Compared with the AEM, the ionomer binder faces a more severe and complex working environment. The cations of ionomer were more easily attacked by OH^–^ as compared with the polumeric backbone.^[^ ^5^ ^]^ To evaluate the stability of the ionomer binder in a real operation environment, ex situ Fourier‐transform infrared (FTIR) and NMR spectra of the samples were acquired in 2 m NaOH solution, and the results are presented in Figure 6b,c. It is clear that no remarkable new stretching vibration peak arises in the FTIR spectrum. The variation in the stretching vibration peaks corresponding to Ar—O—Ar and aromatic C—C of ionomer backbone at 1188 and 1330 cm^–1^ is negligible after 192 h in extremely alkaline solution at 80 °C. Further, no obvious change happens in the peak corresponding to C—N of pyrrolidine at 1462 cm^–1^, indicating that the ionomer can maintain a stable state under a harsh alkaline condition. Meanwhile, no significant change is observed in the NMR spectrum also after 192 h test. Only two minor signals are observed at 1.4 and 2.3 ppm (pink arrow in Figure 6c), whose origin is unknown.^[^ ^47^ ^]^ According to the spectra, the shape, position, and intensity of the peaks corresponding to the pyrroline cations remain unchanged relative to the aromatic signals during the test, and no evident Hofmann *β*‐elimination happens. The peak of the pyrroline ring at nearly 3.2 ppm (*I*_Py_) is observed to decline over time, which is accompanied with the variation in the peak of aromatic signal at 6.3–6.7 ppm (*I*_Ar_). There is a limited degradation of pyrroline group at the position of benzylic extended side chain (4.6% loss after 192 h) under the regular operation of AEMFC. To evaluate the electrochemical stability of ionomer, the PPO‐7Py7 was immersed in 2 m KOH solution under 80 ℃ with 1 V constant‐voltage. The peak area of the NMR remained constant after 96h, as shown in Figure S13, Supporting Information, indicating that the PPO‐7Py7 showed excellent chemical stability performance same as the pure alkaline condition test.

### 2.7 Single‐Cell Performance and In Situ MEA Resistance Test

**Figure 7:**
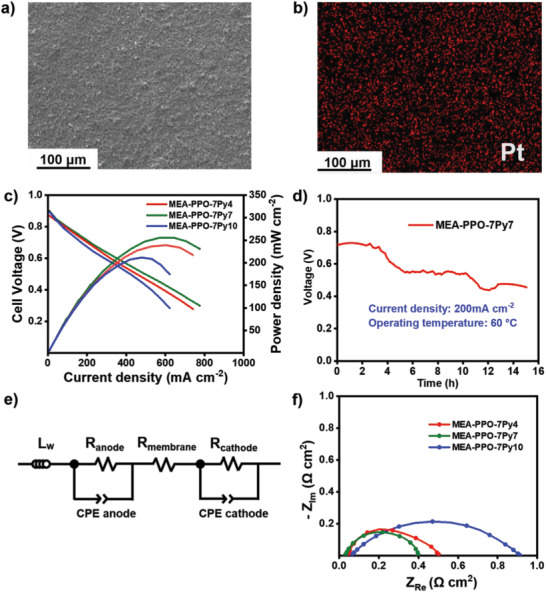
a,b) SEM and EDX analysis of PPO‐7Py7‐based MEA surface, where no obvious agglomeration of Pt catalyst is observed. c) Single‐cell performance of the QPPO membrane with a variety of ionomer binders in H_2_ and O_2_ based on AEMFC tests at 60 °C (anode and cathode: Pt/C; 0.5 mg cm^–2^ of metal loading; 500 mL min^–1^ of gas flow at 100% RH without backpressure). d) Durability of the FC based on the MEA‐PPO‐7Py7 sample, operating at 200 mA cm^–2^ and 60 °C with 0.5 L min^–1^ of gas flow under 100% RH. e,f) Equivalent circuit of AEMFC, and EIS measurements of different MEAs. The MEA‐PPO‐7Py7 sample presents the lowest ohmic resistance, which may be attributed to the fact that a well‐dispersed CL facilitates a lower interface contact resistance.

Finally, for evaluating the performance of PPO‐7Pyn ionomer binder (*n* = 4, 7, 10) at the specific anode and cathode, the quaternized poly(2,6‐dimethyl‐1,4‐phenylene oxide) (QPPO) membrane was utilized as the separator in a single H_2_/O_2_ AEMFC. The ionic conductivity of QPPO membrane is shown in Figure S10, Supporting information. To highlight the dispersion in the CL, the surface of MEAs based on PPO‐7Py7 ionomer were examined using scanning electron microscopy (SEM) and energy dispersive X‐ray (EDX) analysis. As shown in **Figure** **7**a,b, no obvious deficiency and agglomeration of metal catalyst is observed in the SEM and EDX images due to the formation of a well‐dispersed CL, where multiple effects are regulated in the ionomer binder.

All the FCs were operated at 60 °C under completely humidified condition, and the open‐circuit voltages (OCVs) of FCs with MEA‐PPO7Py4, MEA‐PPO7Py7, and MEA‐PPO7Py10 were 1.03, 1.04, and 1.01 V, respectively, which are close to the theoretical value (1.23 V). This confirms that MEAs are gas‐tight assemblies.^[^ ^48^, ^49^ ^]^ The ionomer binder PPO‐7Py7‐modified MEA sample exhibits the best electrochemical performance with the highest peak power density of 261 mW cm^–2^ when the current density is 650 mA cm^–2^ at 0.4 V, which is higher than that of the PPO‐7Py4‐modified MEAs (243 mW cm^–2^ with the current density of 600–610 mA cm^–2^ at 0.45 V), as shown in Figure 7c. The PPO‐7Py10‐modified sample shows the lowest peak power density of 217 mW cm^–2^ and a current density of 490–500 mA cm^–2^ at 0.45 V, which can be attributed to the poor dispersion of catalyst particles and lower ion exchange capacity (IEC) that limit the transport of hydroxide ions in the CL.

Next, to assess the long‐term operational stability of the unique ionomer‐binder‐modified FCs, a durability test was conducted on the MEA‐PPO‐7Py7, which exhibited the best catalytic performance under 100% RH condition. In this test, the variation in the FC voltage was recorded under a constant current density of 200 mA cm^–2^ at 60 °C. As shown in Figure 7d, during the first 3 h of the test, the voltage of cell is maintained at nearly 0.73 V. In the following 2 h, the cell voltage is rapidly decreased from 0.73 to 0.58 V, which may be caused by severe swelling of QPPO membrane under a high RH environment (water uptake: 172% swelling ratio: 38.4% in 60 °C). Then, the voltage of cell becomes relatively stable at ≈0.58 V for 5 h. The stable power output in this device can be attributed to balanced water transportation at the interface of the MEAs as the well‐dispersed CL prevents flooding after the membrane is completely swollen. On the other hand, the MEAs modified by PPO‐7Py7 can maintain a continuous electrochemical reaction because the ionomer binder displays a stable state under the sustained electrochemical environment. Next, the obvious voltage drop was observed after 12 h due to the accumulation of water in the cell. So, we increased the flow rate of the gas briefly to remove the excess water and the voltage of cell gradually back to 0.45 V. Unfortunately, the durability of this cell was still unsatisfactory for a prolonged operation (consistent operation over 15 h), and there exist numerous factors that govern the life of the device, including the MEA fabrication process, the dew point temperature controlled in cells, the degradation of the catalyst, and so on, which should be focused on in the future studies.^[^ ^50^, ^51^, ^52^, ^53^ ^]^

To further examine the surface of each MEA, in situ electrochemical impedance spectroscopy (EIS) was conducted at their OCV under FC operation, and the results are shown in Figure 7e,f. The EIS data are fitted with the equivalent circuit of AEMFCs, as shown in Figure 7e. The high‐frequency intercept (*Z*_Re_) represents the ohmic resistance, which mainly includes the interface contact resistance and the membrane resistance.^[^ ^54^, ^55^ ^]^ Furthermore, the MEAs formed by various ionomer binders (PPO‐7Py4, PPO‐7Py7, and PPO‐7Py10) show comparable ohmic resistances of 0.032, 0.028, and 0.062 Ω cm^2^, respectively, in Figure 7f. The MEA based on PPO‐7Py7 exhibits the lowest non‐ohmic resistance which corresponds to the contact resistance between CL and membrane (the second intercept of the Nyquist plot); the phenomenon may be attributed to a well‐dispersed CL. Besides, the semicircle diameter of the high‐ and low‐frequency arc indicates charge–transfer resistance relative to the electrochemical reaction.^[^ ^56^ ^]^ The MEA‐PPO‐7Py7 sample shows a lowest charge transfer resistance, indicating a rapid electrochemical reaction in the FC and a higher power density. The well‐dispersed CL facilitates rapid ion transport and lower contact resistance. These results further highlight that the ionomer structure with multiple force regulation between the ionomer binder and catalyst particles is conducive to enhance the electrochemical activity.

## 3 Conclusion

In this study, we proposed a new concept of structural evolution of the ionomer binder to regulate the interaction between the ionomer binder and catalyst. A reasonable length of flexible spacer units (seven carbon atoms) between the ionomer backbone and cation mitigated the toxicity of catalyst due to the strong adsorption of ionomer backbone on the metal catalyst. Meanwhile, a rotional adsorption energy (−4.33 eV) between the pyrrolidium cations (terminal alkyl with seven carbon atoms) and catalyst was observed, which promoted the rapid transportation of oxygen, water, and active substrates in the TPB of CL. Therefore, the resultant PPO‐7Py7 ionomer‐binder‐modified CL exhibited homogeneous microtopography and excellent electrochemical performance. Both experimental and computational studies demonstrated that the strategy of interaction regulation alleviates the catalyst agglomeration and facilitates the formation of a state‐of‐the‐art CL in the AEMFCs. To evaluate the electrochemical stability of the cation groups, a constant voltage was applied to the base electrolyte solution for measuring the alkaline stability of cation groups. The degradation of the cation groups tended to accelerate under an electrochemical environment. Overall, the proposed strategy of structural evolution of the ionomer boosts the potential of catalytic reaction in AEMFCs, and the method to measure the durability of ionomer can serve as a useful guide for the development of efficient energy transition devices that include anion conducting electrolytes (water electrolysis, fuel cells, redox flow batteries, etc.).

## 4 Experimental Section

### Materials

Poly (2,6‐dimethyl‐1,4‐phenylene oxide) (PPO, *M*_w_: 10 000 Da) was produced by Asahi Kasei Chemicals Corporation (Japan). *N*‐Bromosuccinimide (NBS), 2,2′‐azobis‐isobutyronitrile (AIBN, 99%, recrystallized from hot ethanol), tetrahydropyrrole, trimethylamine ethanol solution (33%), methyl pyrrolidine, *N*‐butyl bromide, *N*‐heptane bromide, *N*‐decane bromide, *n*‐Butylithium (*n*‐BuLi, 2.5 m, solution in hexane), 1,4‐dibromobutane (99%), 1,6‐dibromohexane (99%), and 1,8‐dibromooctane (99%) were purchased from Aladdin (Shanghai, China). *N*‐Methyl‐2‐pyrrolidolone (NMP, AR), chlorobenzene, tetrahydrofuran (THF), ethanol, methanol, isopropanol, ether, and sodium hydroxide (AR) were purchased from Sinopham Chemical Reagent Co. Ltd. THF was dried by distillation. The Pt/C catalyst (60 wt% platinum on Vulcan XC‐72 carbon support) was supplied by Johnson Matthey (made in USA). Deionized water was filtered by Millipore system with 18.2 MΩ cm^−1^.

### Characterization

^1^H NMR experiments were recorded on a Varian 400 MHz spectrometer. Thermogravimetric analyzer (TGA) analysis was carried out from 30 °C to 800 °C in N_2_ atmosphere by a Universal Analysis 2000, heating rate: 10 °C min^–1^ and the decomposition temperature (*T*_d,95_) of polymer was determined at 5% weight loss. The morphologies of the samples were performed by field‐emission scanning electron microscopy (FESEM Hitachi S‐4700) fitted with an energy dispersive spectrometer (EDS) detector via HRTEM (FEI Tecnai G2 F20). The infrared spectrum of ionomer was measured by a PerkinElmer FT‐IR Spectrum 6700. The contact angles test was gauged by static contact angle measurements via a drop shape analysis system DSA10 (KRUESS, Germany).

### Synthesis of *N*‐Alkyl Pyrrolidine

The variety of alkyl chain pyrrolidines was obtained from dehydrogenate reaction; here *N*‐butyl pyrrolidine synthesis was taken as an example, a mixture containing tetrahydropyrrole (5.0 g, 70 mmol), 25 mL acetonitrile and sodium hydroxide (3.37 g, 84 mmol) was stirred at room temperature for 6 h. Then 1‐bromobutyl (12.35 g, 84 mmol) was added dropwise in the above solution and stirred overnight. After the removal of solvent, the compound was washed several times with deionized water and extracted by dichloromethane to get the final product. The ^1^H NMR spectrums of *N*‐alkyl pyrrolidine are given in Figures S1–S3, Supporting Information.

### Synthesis of BPPO

BPPO was synthesized following the method reported:^[^ ^57^ ^]^ PPO (5.0 g, 41.5 mmol) was dissolved in 100 mL chlorobenzene solution, and then NBS (5.25 g, 29.2 mmol) and AIBN (0.05 g, 0.31 mmol) were added under N_2_ condition. The reaction solution was heated to 135 °C for 3 h with stirring. After the reaction was cooled down, the mixture was poured into excess ethanol (1 L) to form a light‐brown fibrous precipitate product. The precipitate was then washed by ethanol more than three times to get a light‐brown powder. The product was dried in a vacuum oven at 45 °C for 24 h. The ^1^H NMR spectrum of BPPO is given in Figure S4, Supporting Information.

### Synthesis of QPPO

BPPO (3.0 g, 31.32 mmol, 40 degree of bromination) was dissolved in 30 mL of NMP at 40 °C. Then, trimethylamine ethanol solution (2.5 mL, 33 wt%) was added and stirred for 24 h. The mixture was dripped into diethyl ether forming a paint yellow precipitate. The collected product was washed more than three times by ether. The ^1^H NMR spectrum of QPPO is given in Figure S5, Supporting Information.

### Synthesis of PPO‐*n*Br

The expansion of PPO side chain by bromoalkylate lithiation was conducted following the method reported.^[^ ^58^ ^]^ Via the reaction with 1, 4‐dibromobutane, 1, 6‐dibromohexane or 1, 8‐dibromooctane to prepare polymers with a degree of bromination of 40% (DB, percentage of bromoalkylated benzylic methyl groups of the PPO). The samples were denoted as PPO‐*n*Br where *n* (*n* = 5, 7, 9) was the number of carbon atoms in the side chain. The ^1^H NMR spectra of PPO‐5Br, PPO‐7Br, and PPO‐9Br are given in Figures S6–S8, Supporting Information.

Here, the synthesis of PPO‐7Br was taken as an example. The dried PPO (3.0 g, 25.0 mmol) powder was degassed with nitrogen for at least six evacuation cycles and then dissolved in dehydrated THF (300 mL) via syringe under nitrogen condition and stirred at 50 °C to form a homogeneous solution. While waiting for the reactor system to cool down to room temperature, added a few drops of *n*‐BuLi solution to titrate the remaining trace of impurities until a pale gold color persisted to indicate the start of the lithiation reaction. Next, 20.5 mL *n*‐BuLi solution (51.25 mmol) was added to the mixture kept at least 3 h to get a light orange color suspension due to lithiated PPO forming a fine precipitate. Afterward, the mixture was heated to 50 °C again to make all polymers completely dissolved and form a red homogeneous solution. Then, the mixture was cooled to −78 °C via a dry ice/acetone bath. About 200% molar excess of 1, 6‐dibromohexane was injected swiftly to extinguish the lithiated sites on the PPO. A pale gold color solution was instantly formed which was kept overnight at room temperature. The clear solution was collected after a high‐speed centrifugal operation, then, added dropwise to 900 mL methanol to precipitate the white product. Then, the mixture was filtered, collected product, washed repeatedly three times with methanol at least, and dried in 40 °C vacuum oven for over 48 h.

### Synthesis of PPO‐*n*Py*m* and PPO‐*n*QA

The ionomer binder was designated as PPO‐*n*Py*m* and PPO‐*n*QA where *n* is the number of carbon atoms in the side chain spacer close benzene ring and *m* is the outer end chain linked by pyrrolidine cationic. The ionomer binders were prepared via Menshutkin reaction of PPO‐*n*Br and *N*‐alkyl pyrrolidine or trimethylamine. All the polymer ionize processes proceeded with 5 wt% NMP solution at 80 °C for 3 days, obtained from precipitation and purified in ether solvent. The ionization degree of all samples was verified via ^1^H NMR spectra employing DMSO‐d_6_ (*δ* = 2.50 ppm) solutions as shown in Figures S6–S9, Supporting Information.

### AEMs Preparation

QPPO in the as‐synthesized Br^–^ form was dissolved in NMP (7.5 wt%). The solution was filtered via a syringe filter (0.22 µm), cast on a flat square glass plate (0.01 m^2^) and heated at 60 °C for 4 h on a heat plate and then dried in vacuo at room temperature for 12 h, forming films with the thickness of ≈36 µm. The resulting AEMs were peeled from glass by immersing in warm deionized water, then it was immersed in an N_2_‐saturated 1 m NaOH solution at 80 °C for 12 h to convert the membrane from Br^–^ to OH^–^ form, and then thoroughly washed with deionized water.

### Preparation and Electrochemical Testing of Catalyst Inks

The catalyst inks for ORR test were prepared by ultrasonically mixing 4 mg of commercial Pt/C catalyst powder with a mixture including 800 µL isopropanol, 200 µL H_2_O, and 20 mg of PPO‐*n*Py*m* or PPO‐*n*QA ionomer/NMP solution (5 wt%) for 30 min in the ice bath, then took 7 µL inks to drip onto a glassy carbon rotating disk electrode (GC‐RDE, 0.196 cm^2^) three times and dried in room temperature as a working electrode, keeping Pt loading greater than 0.25 mg cm^–2^. A platinum wire and saturated Ag/AgCl electrode were used as the counter and reference electrodes, three‐electrode system was tested by CHI 660E electrochemical workstation and rotate system (Pine Research Instruments, USA) with an aqueous KOH (0.1 m) electrolyte. O_2_ was purged in solution over 30 min before LSV measurement, recorded LSV curves from −1.0 to 0.2 V with a scan rate of 10 mV s^–1^ and rotation speed of 1600 rpm. CV curves were measured from −1.0 to 0.2 V with scanning of 50 mV s^–1^ in N_2_‐saturated and CO‐saturated 0.1 m HClO_4_ solution separately. Then the CO_ads_ stripping lines of the samples were obtained and the ECSA value could be calculated, as described in Equations (1) and (2): where *M*_pt_ represents the platinum loading of the working electrode, and the value of *Q*_co‐adsorption_ is decided by the area of the CO stripping charge (*E*) and the scanning rate of CV (*ν*)

(1) $$\mathrm{ECSA}\left(\frac{m^{2}}{g}\right)=\left[\frac{Q_{\mathrm{CO}-\mathrm{adsorption}\left(C\right)}}{420\left(\mu \frac{c}{cm^{2}}\right)M_{\mathrm{Pt}}\left(\mathrm{mg}\right)}\right]10^{5}$$

(2) $$Q_{\mathrm{CO}-\mathrm{adsorption}}\;\left(C\right)=\frac{\int idE\left(\mathrm{mAV}\right)}{v\left(\mathrm{mv}\;s^{-1}\right)}$$

### DFT Computational Details and Analysis

Theoretical adsorption energies of pyrrolidinium cation groups with Pt were performed using Materials Studio (version 8.0). Dmol^3^ available was operated with molecule geometry optimizations.^[^ ^59^ ^]^ All the calculations were operated by double numerical plus polarization (DNP) and generalized gradient approximation with BLYP function (GGA‐BLYP) basis set.^[^ ^60^, ^61^ ^]^

To explore the adsorption energy calculations, the electronic structure calculations were performed using the Cambridge serial total energy package program module (CASTEP).^[^ ^62^ ^]^ The simulated Pt (111) catalyst surface was modeled utilizing four layers of metal atoms with the dimensions of 16.62 × 16.65 Å and an additional vacuum region of 20 Å, resulting in 192 metal atoms in each unit cell. From this reaction, the adsorption energies could be calculated by the following Equation (3):

(3) $$\Delta E_{\mathrm{adsorption}}=E_{\mathrm{Cations}}\;+E_{\mathrm{Pt}}-E_{\left(\mathrm{Pt}+\mathrm{Cations}\right)}$$

where *E*_cations_ was the energy of cations (*N,N*‐dimethyl pyrrolidine, *N*‐butyl‐1‐methyl pyrrolidine, *N*‐heptyl‐1‐methylpyrrolidine, *N*‐decyl‐1‐methylpyrrolidine salt) in the unit cell of 20.00 × 20.00 × 20.00 Å. The *E*_pt_ was the energy of the Pt surface, *E*_(Pt+Cations)_ is the energy of the adsorbed on the surface of the Pt (1,1,1).

### Membrane Electrode Assembly (MEA) Fabrication and Single‐Cell Testing

Pt/C (6.75 mg) catalyst powder was ultrasonically mixed with 250 mg of isopropanol, 62.5 mg of deionized water, and 33.75 mg of ionomer solution (5 wt% PPO‐*7*Py*m* in NMP aqueous) for 1 h in the ice water bath to get a catalyst ink. The prepared catalyst ink was sprayed on a QPPO membrane (1.8 cm × 1.8 cm) to form catalyst layers for both sides which contained 20 wt% ionomers and 80 wt% catalysts and the metal content of each side was controlled to be 0.5 mg_metal_ cm^–2^. Consequently, the catalyst‐coated membrane was immersed into 1 m NaOH solution to exchange hydroxide ions for 2 h and remove residual NaOH by washing with deionized water. Lastly, MEA was assembled as sandwich structures by two match‐sized carbon papers. The fuel cell performance was measured with a flow rate of 500 mL min^–1^ fully humidified hydrogen and oxygen at 60 °C without backpressure.

### MEA In Situ Electrochemical Impedance Spectroscopy (EIS) Test

In situ EIS was measured for each single cell when it operated at its open‐circuit voltage (OCV) with an amplitude of 5 mV, frequency range from 0.1 to 100 kHz. The collected EIS data were fitted by using the Z‐View program (Scribner Associates Inc.)

### Alkaline Stability Test

The evaluation of alkaline stability for ionomer binder was performed by immersing several ionomer membranes (8 mm × 8 mm) in 2 m aqueous. NaOH at 80 °C. The soaked membranes were extracted after 48, 96, and 192 h, recorded by FT‐IR spectrum, each sample ion exchanged to Br^–^ form then dissolved in DMSO‐d6, measured by ^1^H NMR spectroscopy to determine whether a possible polymer structure degradation existed.

## Conflict of Interest

The authors declare no conflict of interest.

## Supporting information

Supporting Information

## Data Availability

All the data needed to evaluate the conclusions in the paper are presented in the paper and/or the Supporting Information. Additional data related to this paper may be requested from the authors.
