# Supplementary material for: Interaction Regulation Between Ionomer Binder and Catalyst: Active Triple‐Phase Boundary and High Performance Catalyst Layer for Anion Exchange Membrane Fuel Cells
Source: Adv Sci (Weinh). 2021 Aug 2;8(19):2101744. doi: 10.1002/advs.202101744 (PMC8498875; doi:10.1002/advs.202101744)
Supplement: Supplementary file 1 — Supporting Information [file ADVS-8-2101744-s001.pdf]

© 2021 Wiley-VCH GmbH

ADVANCED SCIENCE
Open Access

Supporting Information

for *Adv. Sci.*, DOI: 10.1002/advs.202101744

Interaction Regulation Between Ionomer Binder and Catalyst: Active Triple-phase Boundary and High Performance Catalyst Layer for Anion Exchange Membrane Fuel Cells

Huixing Cao, Ji Pan, Hairong Zhu, Zhe Sun, Bowen Wang, Junliang Zhao, Feng Yan,**

© Copyright 2020. WILEY-VCH GmbH.

Supporting Information

Interaction Regulation Between Ionomer Binder and Catalyst: Active Triple-phase Boundary and High Performance Catalyst Layer for Anion Exchange Membrane Fuel Cells

Huixing Cao, Ji Pan, Hairong Zhu, Zhe Sun, Bowen Wang, Junliang Zhao, Feng Yan,**

Prof. Dr.F. Yan, H. Cao, Dr. J. Pan, H. Zhu, Dr. Z. Sun, B. Wang, J. Zhao
College of Chemistry, Chemical Engineering and Materials Science
Soochow University, Suzhou, 215123, China
E-mail: zsun@suda.edu.cn (Z. S.); or fyan@suda.edu.cn (F. Y)

1. ^{1}H NMR spectroscopic characterization

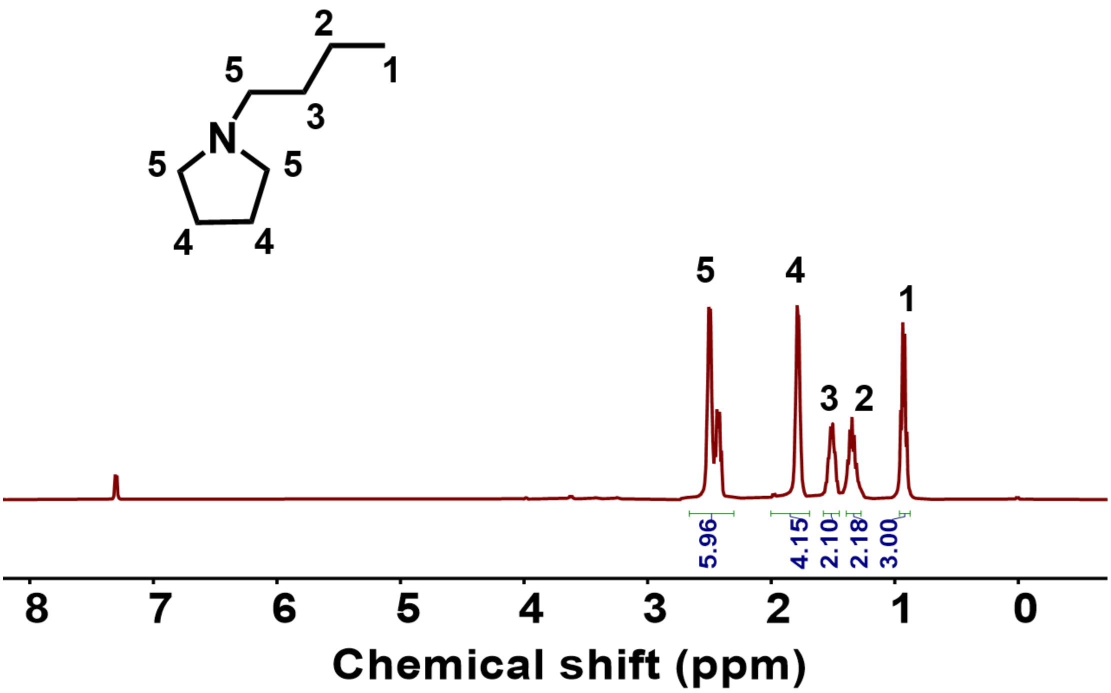

Figure S1. ^{1}H NMR spectrum of butyl-pyrrolidine using $CDCl_3$ as solvent

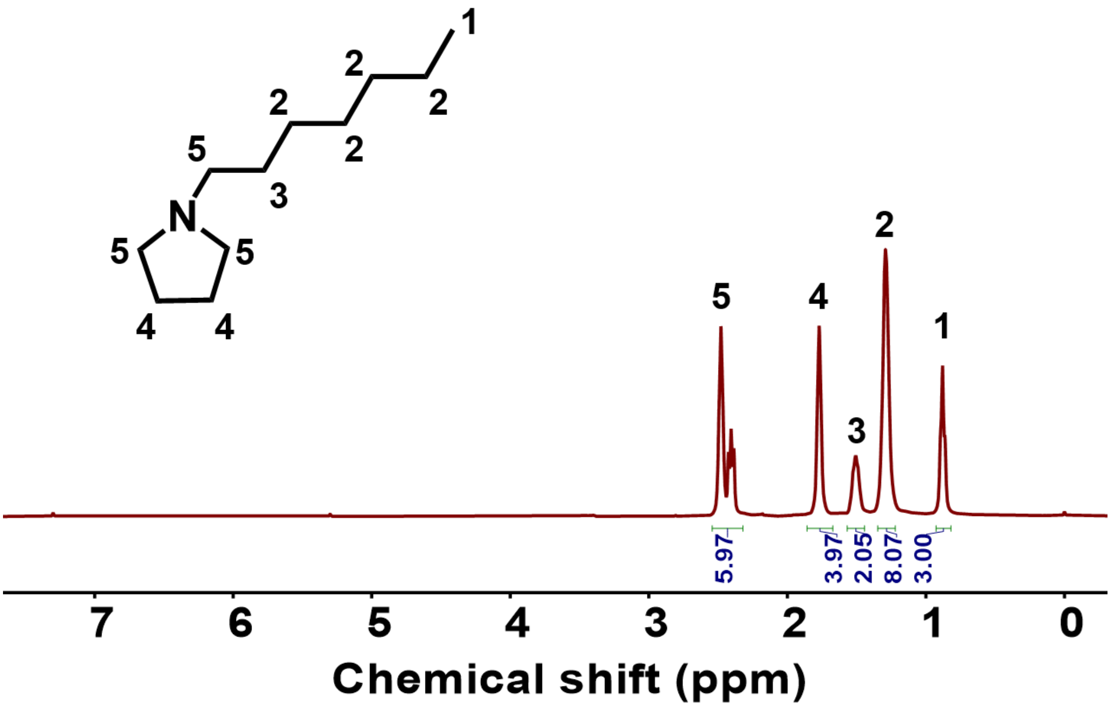

Figure S2. ^1H NMR spectrum of heptyl-pyrrolidine using $CDCl_3$ as solvent

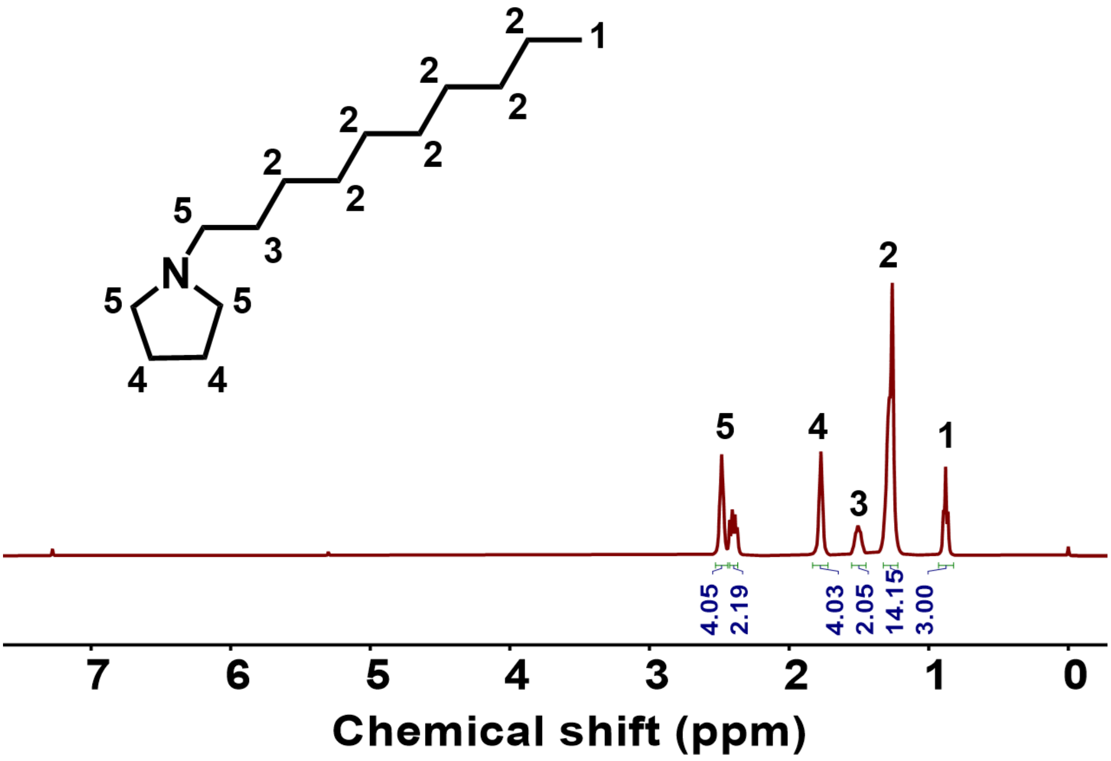

Figure S3. ^1H NMR spectrum of decyl-pyrrolidine using $CDCl_3$ as solvent

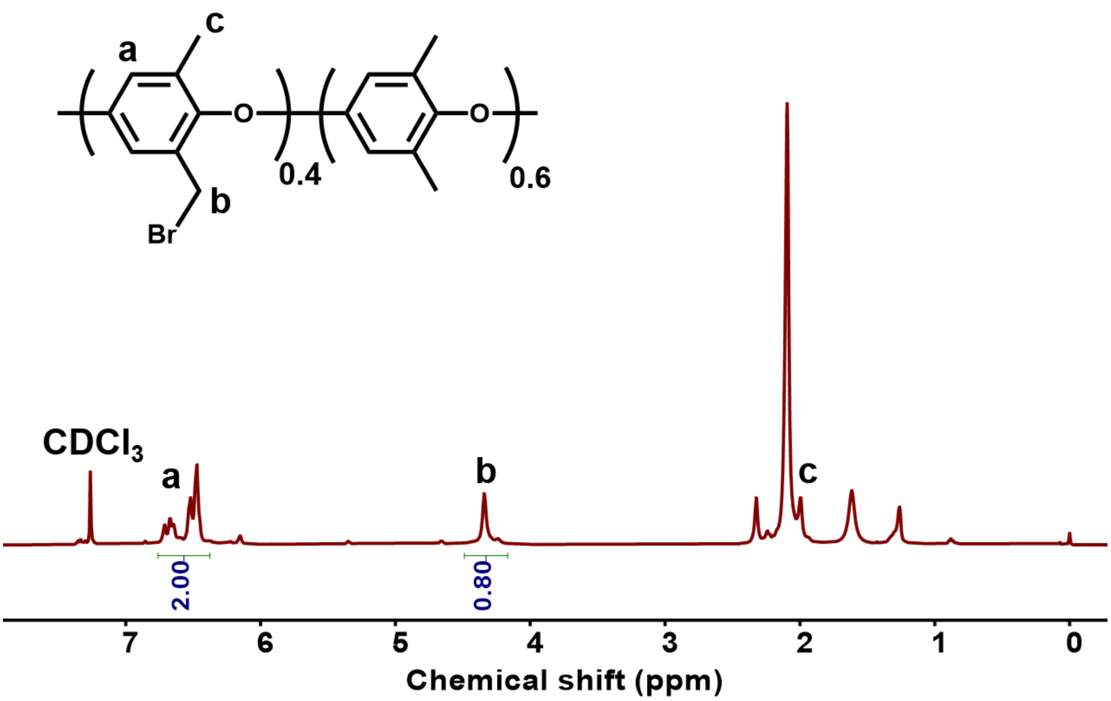

Figure S4. ^{1}H NMR spectrum of BPPO using $CDCl_3$ as solvent

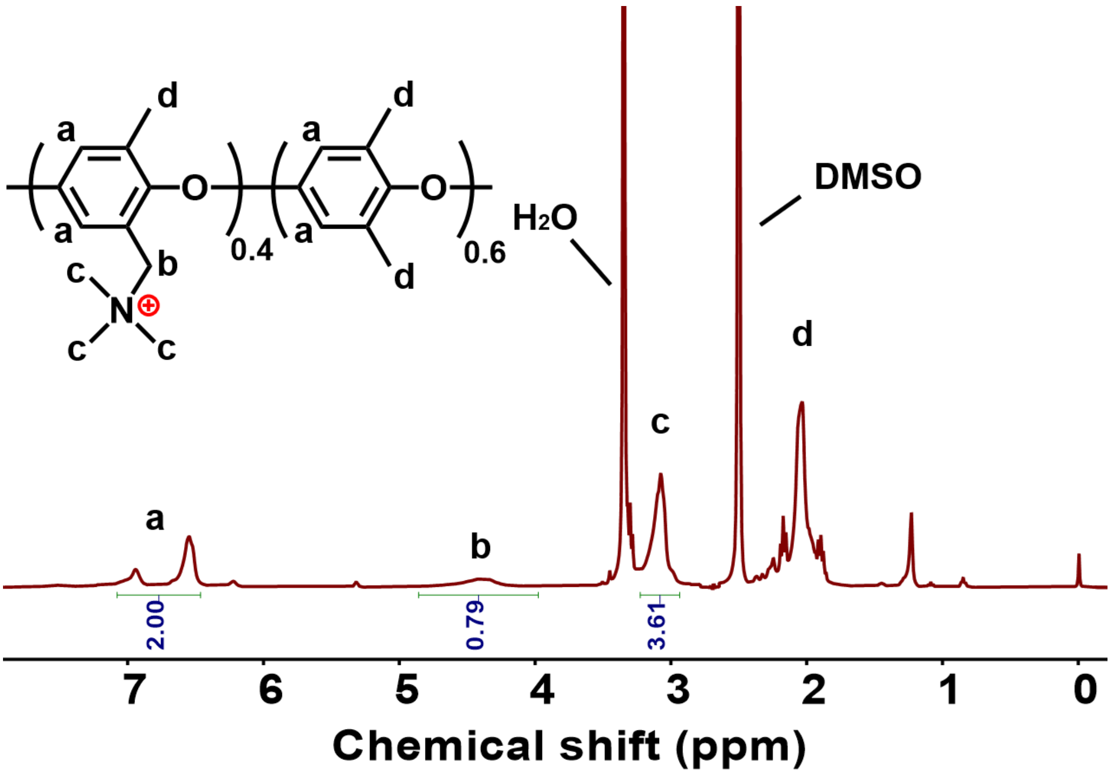

Figure S5. ^{1}H NMR spectrum of QPPO using DMSO-d_6 as solvent

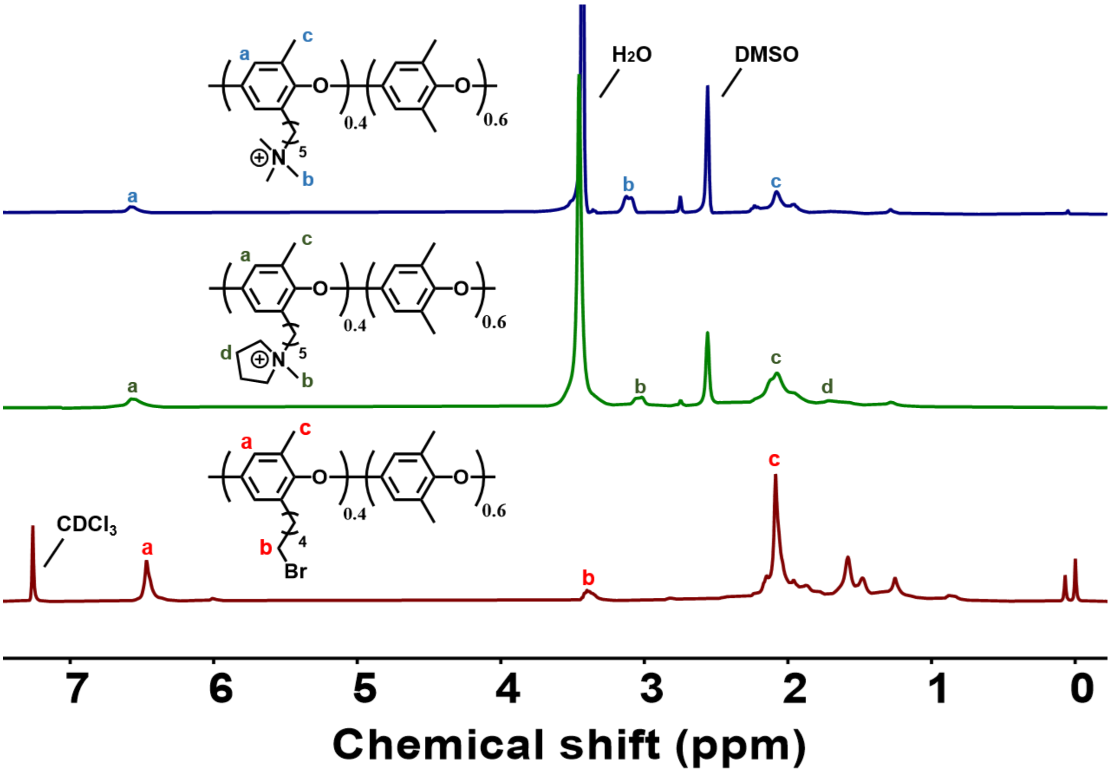

Figure S6. ^{1}H NMR spectrum of PPO-5QA, PPO-5Py using DMSO-d_6 and PPO-5Br using $CDCl_3$ as solvent.

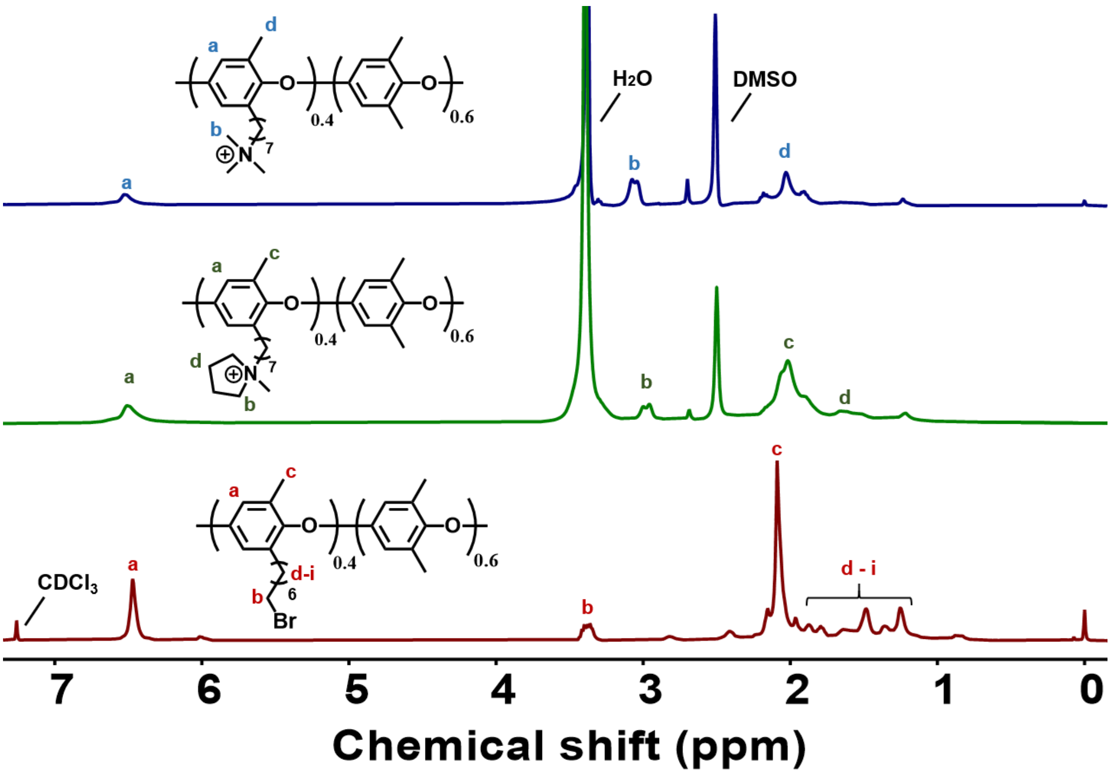

Figure S7. ^{1}H NMR spectrum of PPO-7QA, PPO-7Py using DMSO-d_6 and PPO-7Br using $CDCl_3$ as solvent.

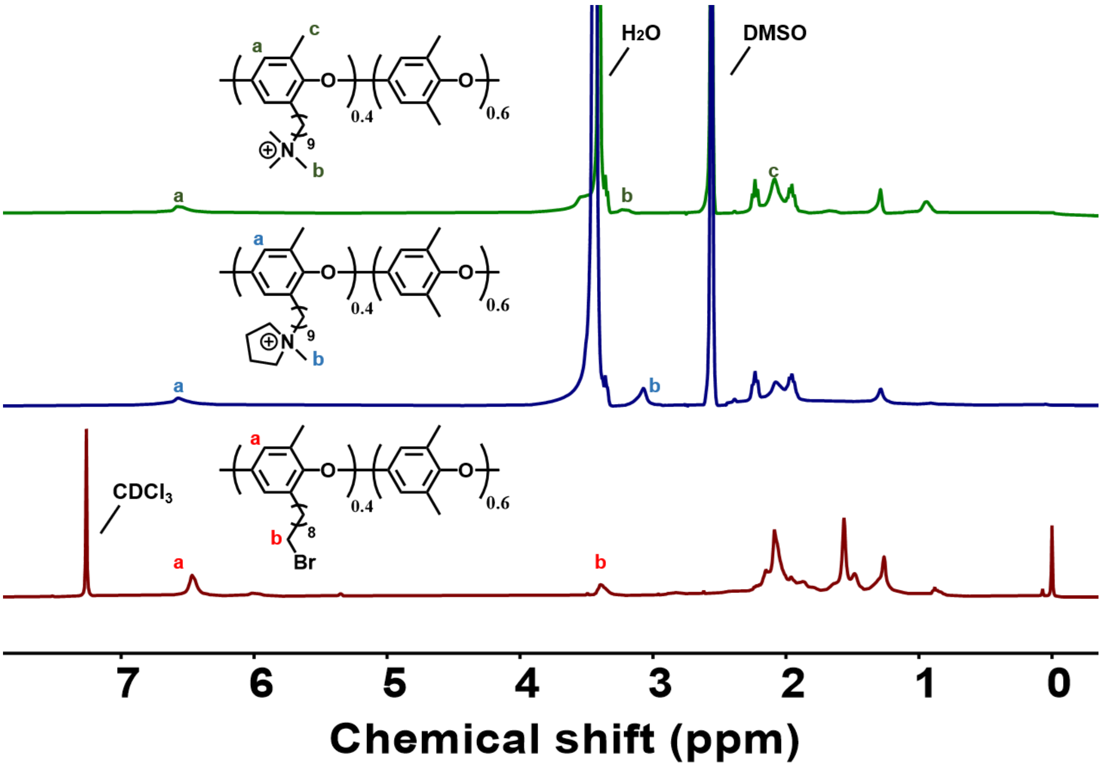

Figure S8. ^{1}H NMR spectrum of PPO-9QA, PPO-9Py using DMSO-d$_6$ and PPO-9Br using $CDCl_3$ as solvent.

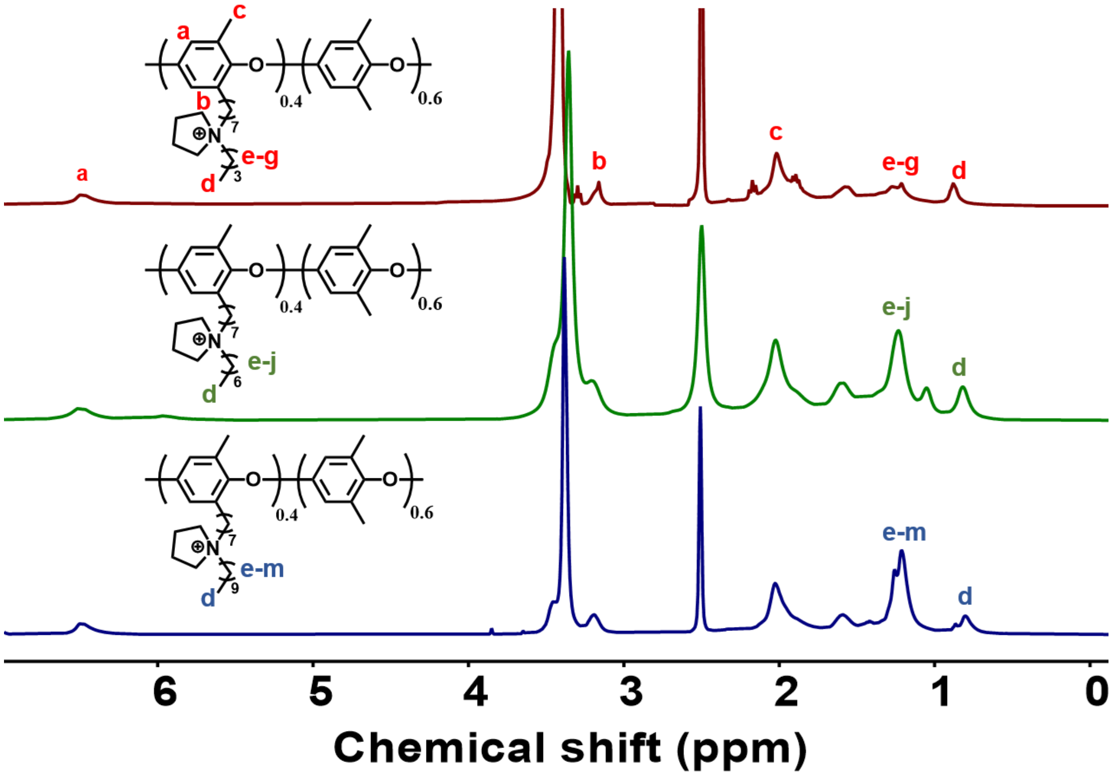

Figure S9. ^{1}H NMR spectrum of PPO-7Py4, PPO-7Py7 and PPO-7Py10 using DMSO-d$_6$ as solvent.

2. AEM test

water uptake (WU) and swelling ratio (SR) test

The wet membrane with the OH^- form (soak in 1 M NaOH aqueous solution 12 h then washed by deionized water 6 times, after being soaked in DI water for 12 h at room temperature and surface water removed) was measured for weight (W_{wet}) and in-plane size (D_{wet} and W_{wet}). Then the membrane was vacuum dried at 25 ℃ overnight; its weight (W_{dry}) and in-plane size (D_{dry} and L_{dry}) were recorded again. SR and WU were calculated by the following formula equation (1) and (2):

$$\mathrm{WU}\ (\%) = \frac{W_{wet} - W_{dry}}{W_{dry}} \times 100\% \quad (1)$$

$$\mathrm{SR}\ (\%) = \frac{L_w - L_d}{L_d} \times 100\% \quad (2)$$

Ion exchange capacity (IEC) of ionomer binder or membrane:

The wet OH^- form ionomer states or membrane sample was soaked into 10 mL of 0.01 M HCl solution for 12 h at room temperature to avoid carbonation. The above solution was titrated with 0.01 M KOH solution using phenolphthalein reagent as an indicator and the volume of KOH solution was recorded as V_{NaOH}. After washed by DI water, the sample converted to OH^- form and vacuum dried, the weight (W_{dry}) of this sample was recorded. IEC was calculated by the following formula equation (3)：

$$\mathrm{IEC} = \frac{V_{HCl} \times C_{HCl} - V_{NaOH} \times C_{NaOH}}{\mathrm{w_{dry}}} \quad (3)$$

Table S1 Characteristic of IEC value for QPPO membrane and various ionomer binder

Sample	IEC[a] (mmol / g)	IEC[b] (mmol / g)
QPPO	2.57	2.54
PPO-5QA	2.24	2.19
PPO-5Py	2.12	2.06
PPO-7QA	2.11	2.09
PPO-7Py	2.01	1.96
PPO-7Py4	1.85	1.81
PPO-7Py7	1.72	1.65
PPO-7Py10	1.60	1.58
PPO-9QA	2.0	1.98
PPO-9Py	1.89	1.85

[a] IEC was calculated via ^{1}H NMR analysis, [b] IEC was calculated via back titration experiment

Ionic conductivity

In-plane ionic conductivities (σ) of QPPO membrane was measured in deionized water with four-point probe AC impedance spectroscopy in a range of frequency from 1 Hz to 100 Hz. The samples were hydrated in deionized water for 24 h before the measurement. Through-plane conductivity (σ) of the membranes was measured by the two probe AC impedance method. The ionic conductivity can be calculated from the following equation (4):

$$\sigma = \frac{l}{RA} \quad (4)$$

where l represents the distance between two reference electrodes. A is the cross-sectional area of the membrane and R is the membrane resistance.

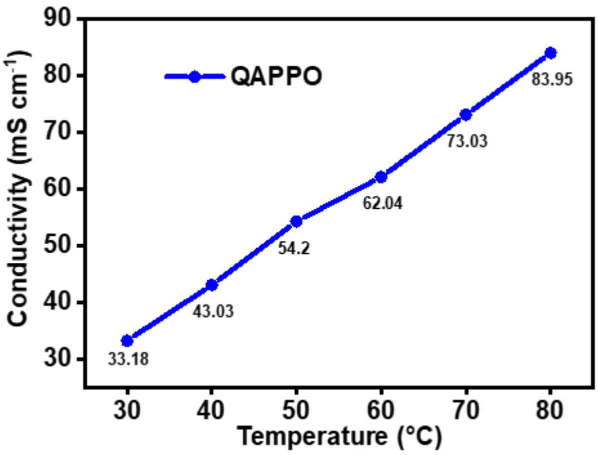

Figure S10. Conductivity plot of QPPO membrane.

3. Electrochemical stability test.

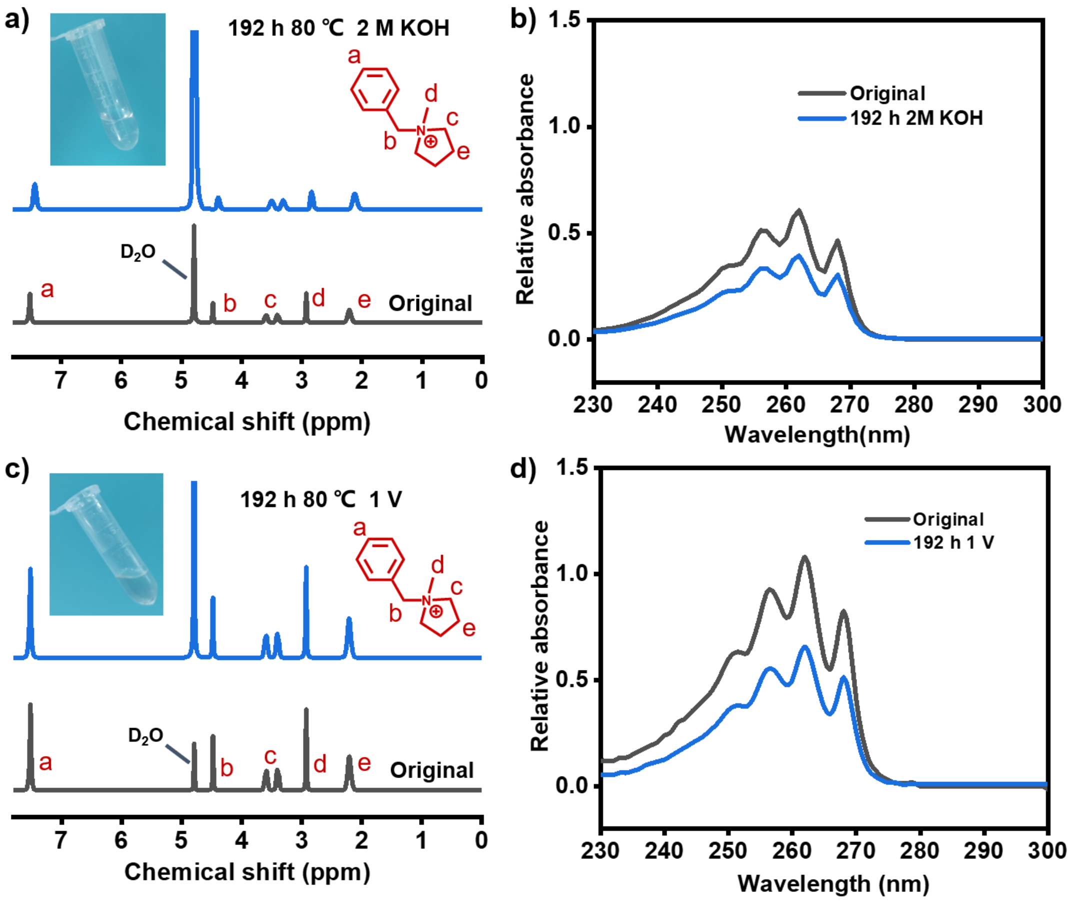

Figure S11. (a-b) NMR and UV spectra of benzyl-methyl pyrrolidine salt in 2 M KOH D_2O electrolyte at 80 °C without constant voltage. No evident variation can be observed after the test. (c-d) NMR and UV spectra of benzyl-methyl pyrrolidine salt under a 1V voltage in D_2O electrolyte at 80 °C. No evident variation can be observed after the test. The results prove that

the constant voltage accelerates the alkaline degradation of the cation groups in the electrolyte, and this effect has been neglected in the earlier studies.

4. CV curves of ionomer binders for ECSA

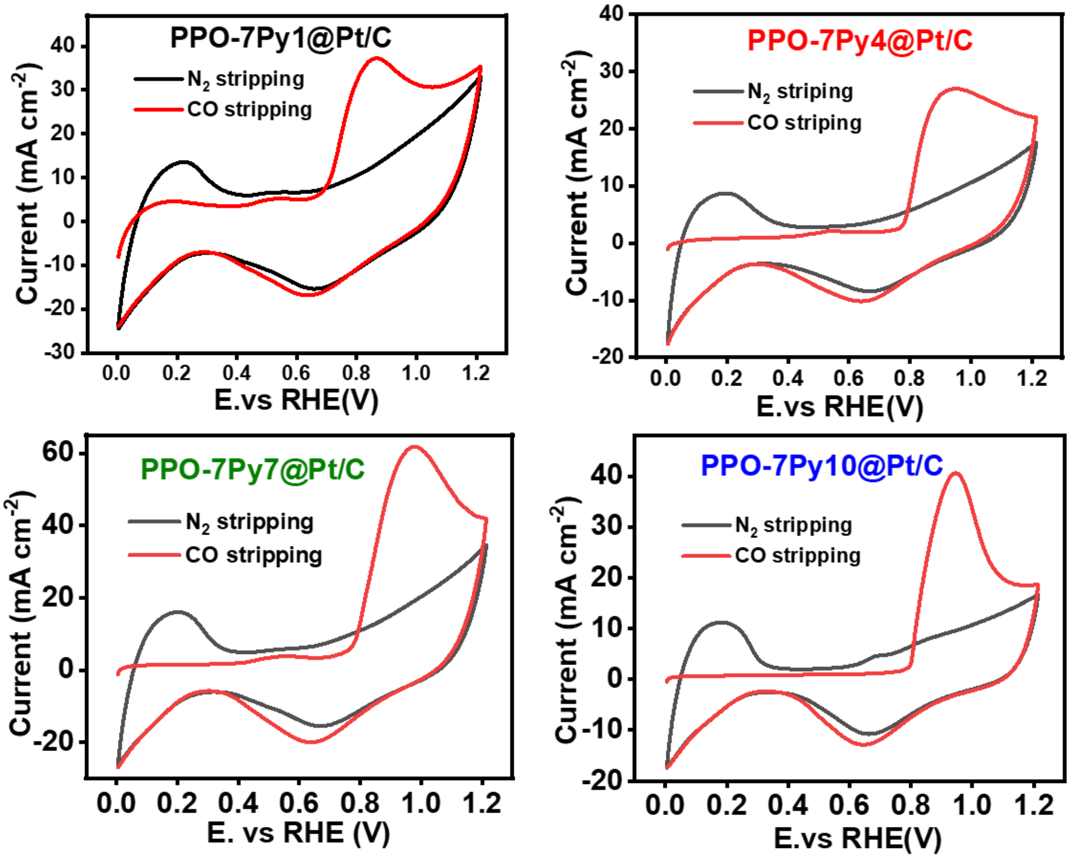

Figure S12 CV curves of various ionomers for ECSA evaluation.

5. Electrochemical stability of ionomer binders

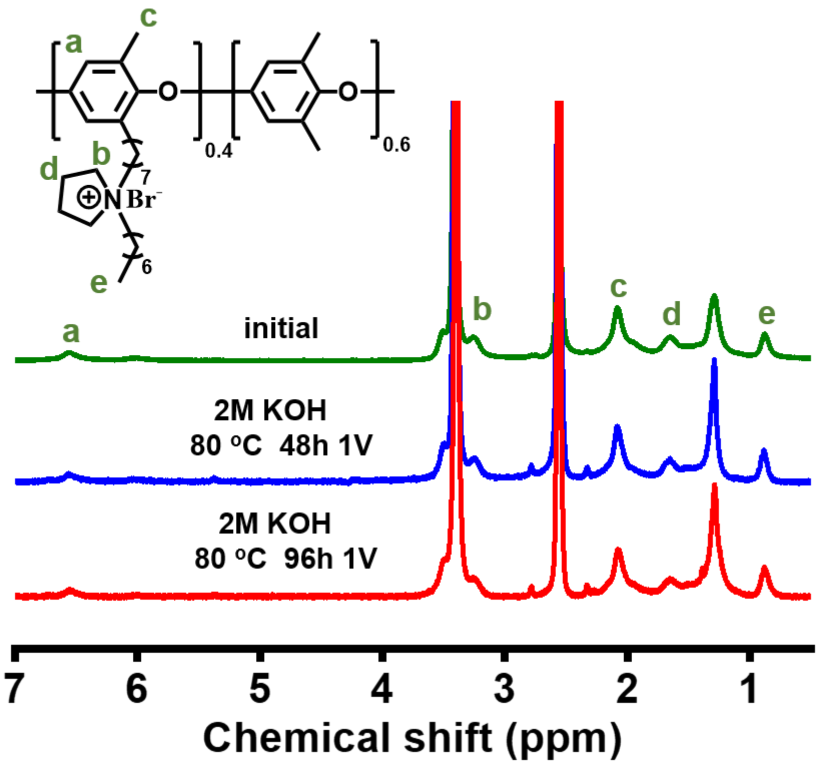

Figure S13 ^{1}H NMR spectra of PPO-7Py7 stored in 2 M aqueous KOH solution at 80 °C with 1 V constant-voltage after 0, 48 and 96 h.
